# Supplementary material for: High output mode-locked laser empowered by defect regulation in 2D Bi2O2Se saturable absorber
Source: Nat Commun. 2022 Jul 5;13:3855. doi: 10.1038/s41467-022-31606-8 (PMC9256711; doi:10.1038/s41467-022-31606-8)
Supplement: Supplementary file 1 — Supplementary Information [file 41467_2022_31606_MOESM1_ESM.pdf]

Supplementary Information for: High output mode-locked laser empowered by defect regulation in 2D Bi_2O_2Se saturable absorber

Junting Liu[1,#], Fang Yang[2,#], Junpeng Lu[2,], Shuai Ye[1], Haowen Guo[1], Hongkun Nie[1], Jialin Zhang[2], Jingliang He[1], Baitao Zhang[1,*], Zhenhua Ni[2,*]*

[1] State Key Laboratory of Crystal Materials, Institute of Novel Semiconductors, Shandong University, 250100, Jinan, Shandong, China

[2] School of Physics and Key Laboratory of MEMS of the Ministry of Education, Southeast University, Nanjing 211189, China

[#] *These authors contributed equally to this work.*

[*] *Correspondence and requests for materials should be addressed to Junpeng Lu (email: phyljp@seu.edu.cn), Baitao Zhang. (email: btzhang@sdu.edu.cn) or Zhenhua Ni (email: zhni@seu.edu.cn)*

Contents:

Supplementary I. Preparation and Characterization of Bi_2O_2Se nanoplates
Supplementary II. Mode-locked laser at 1.04 μm
Supplementary III. Nondegenerate pump-probe measurements
Supplementary IV. TEM analysis of Bi_2O_2Se nanoplates
Supplementary V. Nonlinear optical absorption measurements
Supplementary VI. Improvement of pulsed laser performance
Supplementary VII. Mode-locked laser at 2.0 μm
Supplementary VIII. Reference

Supplementary I. Preparation and Characterization of Bi_2O_2Se nanoplates

The layered Bi_2O_2Se exhibits a tetragonal crystal structure (I4/mmm, a = b = 3.88 Å, c = 12.16 Å, and Z = 2), consisting of $[Bi_2O_2]_n^{2n+}$ and $[Se]_n^{2n-}$ layers bonded by weak intermolecular forces [1, 2], as illustrated in Supplementary Fig. 1a. The thickness of the monolayer containing $[Bi_2O_2]_n^{2n+}$ and $[Se]_n^{2n-}$ layers is 0.61 nm. Larger-area Bi_2O_2Se nanoplates were synthesized via a chemical vapor deposition method (CVD), as shown in Supplementary Fig. 1b. Supplementary Fig. 1c shows that the as-grown Bi_2O_2Se has a square shape with a lateral size up to ~32 µm. The surface of the as-grown Bi_2O_2Se nanoplates, which was determined by atomic force microscopy (AFM), was clean and homogeneous, as shown in Supplementary Fig. 1e, f. The characteristic A_{1g} peak of Bi_2O_2Se can be found at ≈159.4 cm^{-1} in the Raman spectrum, as shown in Supplementary Fig. 1d, which is in agreement with previous studies [3, 4]. In addition, the characterization of the Raman spectra for the same Bi_2O_2Se SA before and after exposure to air for three months demonstrates that the device has excellent long-term stability.

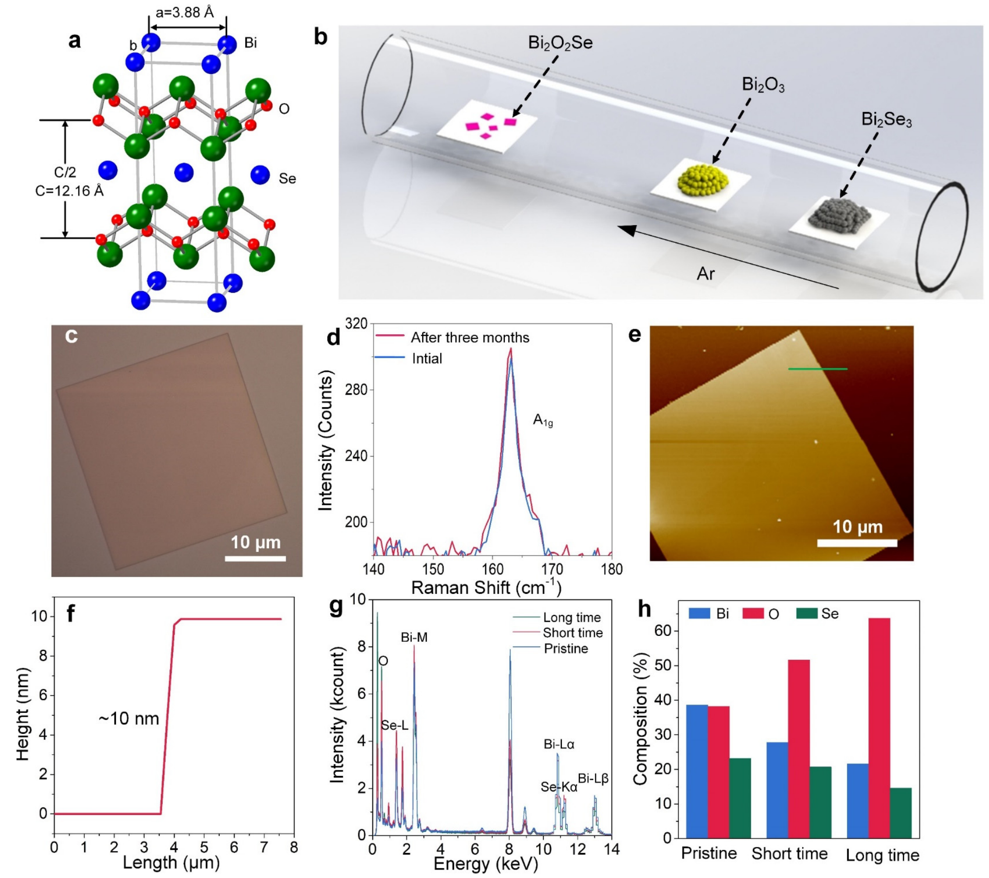

Supplementary Fig. 1 Preparation and Characterization of Bi_2O_2Se nanoplates. a Crystal structure of Bi_2O_2Se with $[Bi_2O_2]_n^{2n+}$ layers and $[Se]_n^{2n-}$ layers. **b** Schematic of the CVD setup for the synthesis of Bi_2O_2Se nanoplates on mica with the sources of Bi_2O_3 and Bi_2Se_3. **c** Typical optical microscopy image of Bi_2O_2Se nanoplates on f-mica. The size is ~32 µm. d Raman spectra of Bi_2O_2Se nanoplates before and after exposure to air for three months. **e** AFM topography of the as-grown Bi_2O_2Se. **f** The corresponding surface roughness. **g** EDS spectra. **h** EDS quantitative analysis of Bi, O and Se in Bi_2O_2Se nanoplates under different argon plasma treatment times.

Supplementary II. Pulsed Laser Applications of Pristine Bi_2O_2Se nanoplates

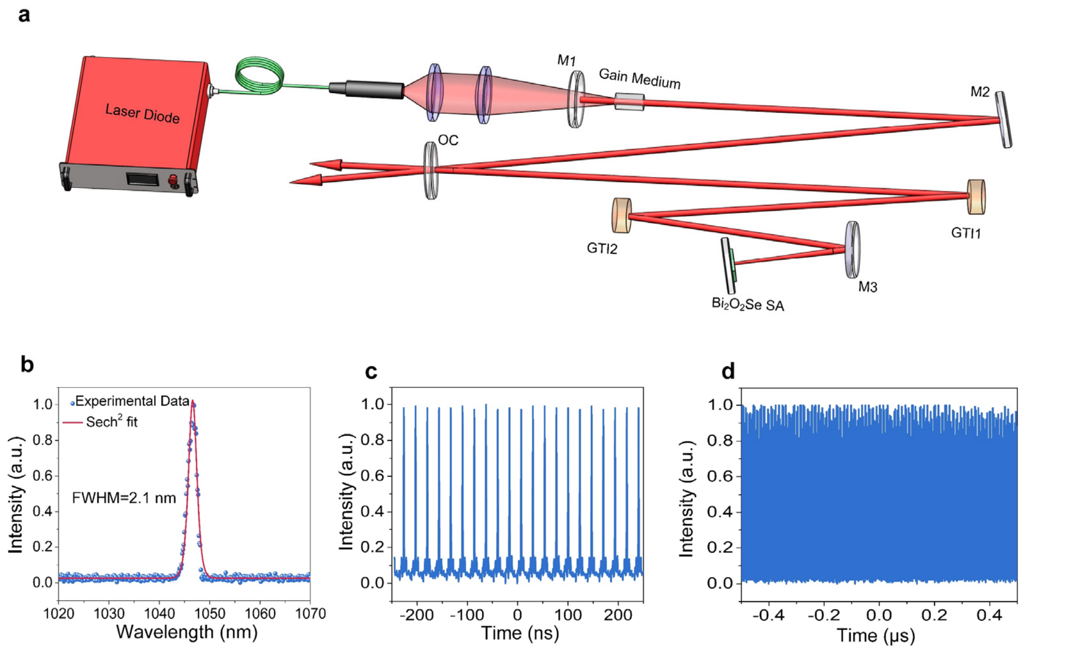

Supplementary Fig. 2 Pulsed Laser Applications of Pristine Bi_2O_2Se nanoplates. a Experimental setup of the mode-locked solid-state bulk laser. **b** The output mode-locked spectrum. **c** The pulse train with a resolution of 50 ns/div. **d** Pulse trains with a resolution of 1 ms/div.

Supplementary III. Nondegenerate pump-probe measurements

Supplementary Table 1. The fitting parameters obtained from a biexponential function to model pump-probe traces of pristine Bi_2O_2Se nanoplates under different input light intensities.

Input light intensity(μJ cm^{-2})	D1	D2	τ_1(ps)	τ_2(ps)
71.9	0.33	0.67	0.56±0.06	106.2±12.2
47	0.35	0.65	1.01±0.08	81.1±1.2
33.2	0.39	0.61	0.91±0.07	122.1±2.7
17.5	0.45	0.55	0.86±0.06	115.1±3.2

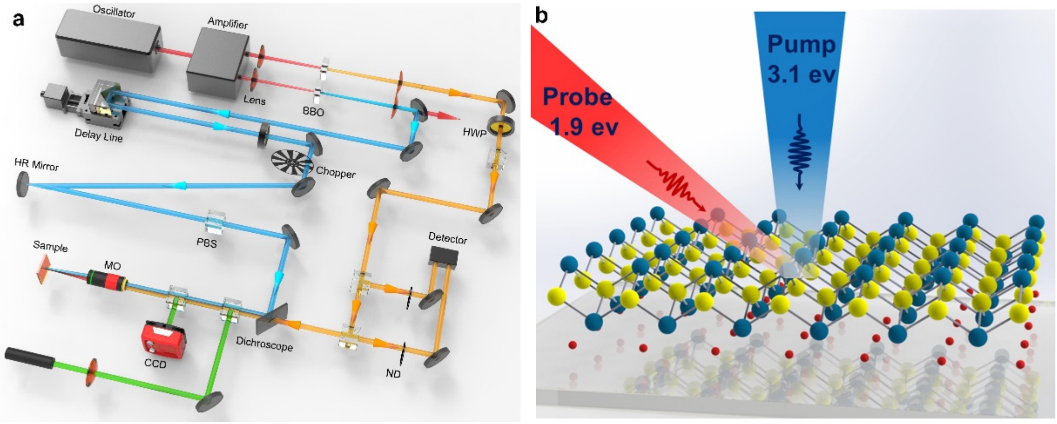

Supplementary Fig. 3 Nondegenerate pump-probe measurements. a Experimental setup of the nondegenerate pump-probe measurement. **b** Schematic illustration of pump-probe spectroscopy.

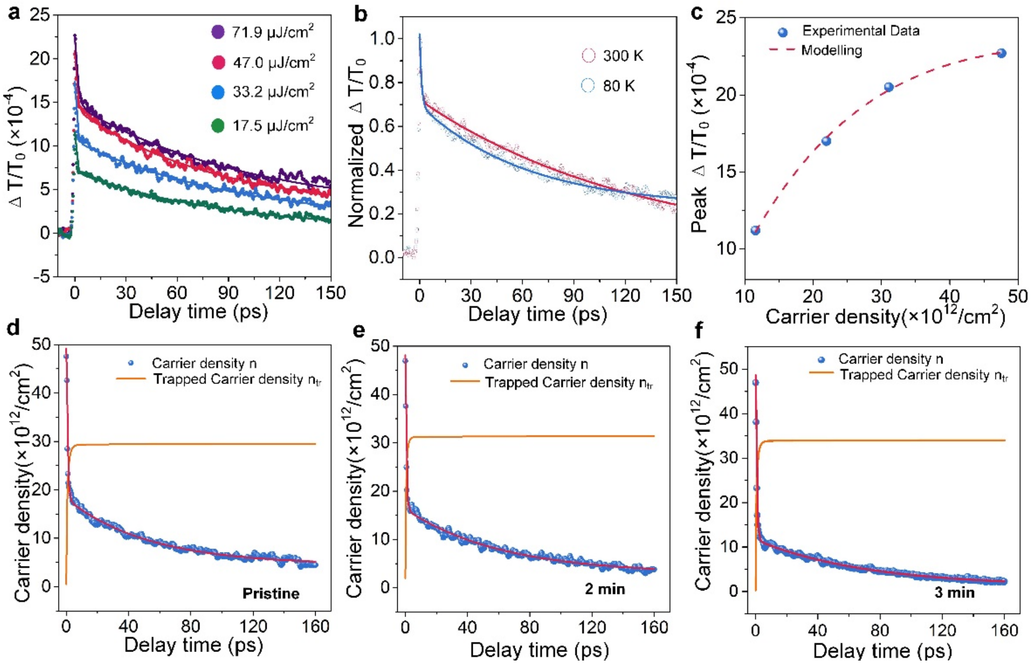

Supplementary Fig. 4 Nondegenerate pump-probe measurements. a The differential transmission of Bi_2O_2Se nanoplates for different pump pulse energies at T=300 K. **b** The differential transmission of Bi_2O_2Se nanoplates for different temperatures. **c** The relationship between the maximum differential transmission and initial photoinduced carrier density. **d-f** The simulation results of Bi_2O_2Se nanoplates under different plasma irradiation times. Blue dots are the time evolution of the carrier density. The red (orange) line indicates the simulated temporal evolution of the free (trapped) carrier density using Equation (1) (2).

Supplementary IV. TEM analysis of Bi_2O_2Se nanoplates

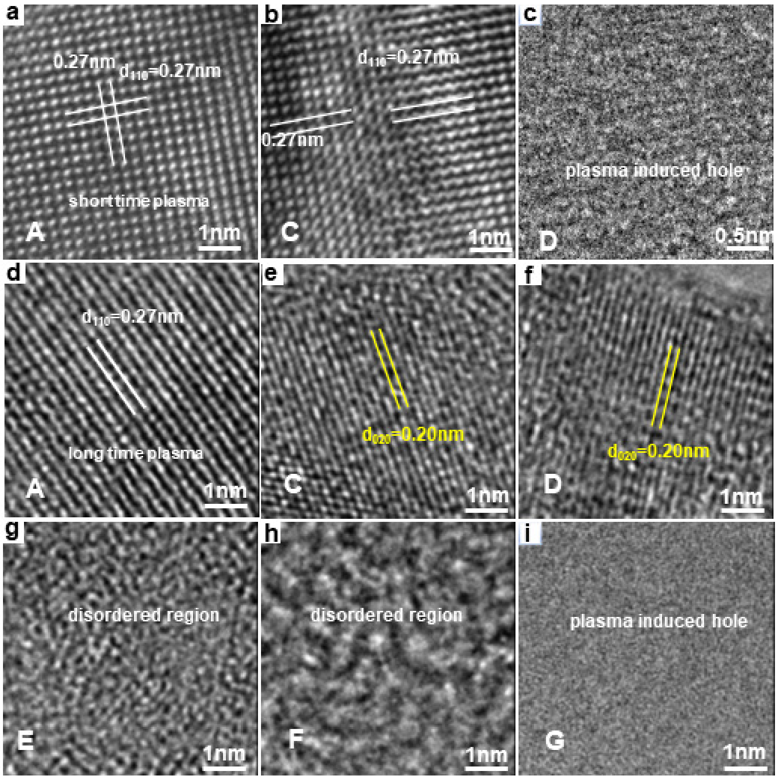

Supplementary Fig. 5 TEM analysis of Bi_2O_2Se nanoplates. a-c Close-up STM images of regions A, C, and D highlighted in panel Fig. 1**d** of the Bi_2O_2Se sample after short-term plasma treatment. **d-i** Close-up STM images of regions A and C-G highlighted in panel Fig. 1**g** of the Bi_2O_2Se sample after long-term plasma treatment.

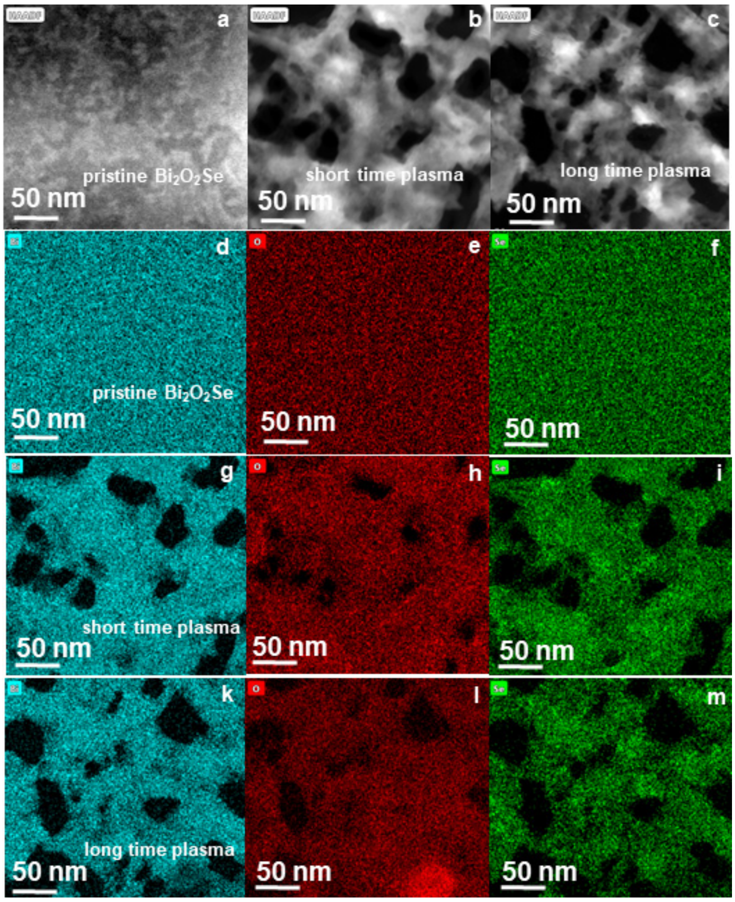

Supplementary Fig. 6 High-angle annular dark-field (HAADF) image of a Bi_2O_2Se nanoplate: a pristine, **b** short-term plasma treatment, and c long-term plasma treatment. **d-f** EDS elemental mapping of Bi, O and Se of the pristine Bi_2O_2Se nanoplates. **g-i** EDS elemental mapping of Bi, O and Se of short-term argon plasma-treated Bi_2O_2Se nanoplates. **k-m** EDS elemental mapping of Bi, O and Se of long-term argon plasma-treated Bi_2O_2Se nanoplates.

Supplementary V. Nonlinear optical absorption measurements

The normalized transmittance can be written as [5]:

$$T(Z) = \sum_{n=0}^{\infty}\left(-\beta_{eff}I_0L_{eff}\right)^n/\left(1+\frac{Z^2}{Z_0^2}\right)^n(n+1)^{3/2}$$

$$= 1-\beta_{eff}I_0L_{eff}/2^{\frac{3}{2}}(1+\frac{Z^2}{Z_0^2}) \quad (1)$$

where β_{eff} is the nonlinear absorption coefficient, $L_{eff}=(1-e^{-a_0L})/a_0$ is the effective thickness of Bi_2O_2Se nanoplates (a_0 is the linear absorption coefficient, L is the thickness of Bi_2O_2Se nanoplates), and z_0 and I_0 are the Rayleigh length and the peak on-axis intensity at the focus (z = 0). Furthermore, the imaginary part of the third-order nonlinear susceptibility $Im\chi^{(3)}$ can be calculated from β_{eff} based on the following formula [6]:

$$Im\chi^{(3)}=\frac{2\varepsilon_0c^2n_1^2}{3\omega}\beta_{eff} \quad (2)$$

where ε_0 is the vacuum permeability, c is the vacuum light speed, ω is the angular frequency, and n_1 is the linear refractive index.

Then, the real part of the third-order nonlinear optical susceptibility ($Re\chi^{(3)}$) can be expressed as [7]

$$Re\chi^{(3)}=\frac{4\varepsilon_0cn_0^2}{3}n_2 \quad (3)$$

In this case, as shown in Supplementary Table 2, the largest values of the calculated $Re\chi^{(3)}$ of pristine Bi_2O_2Se nanoplates are (-1.587±0.009) × 10^{-6} esu.

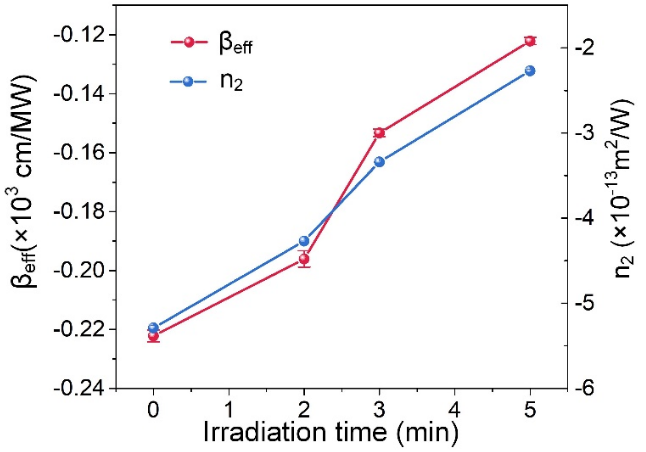

Supplementary Fig. 7 Trend of the effective nonlinear absorption coefficient (β_{eff}) and nonlinear refractive index (n_2) under different plasma irradiation times.

Supplementary Table 2. The fitting parameters obtained from Z-scan characterizations of Bi_2O_2Se nanoplates at different plasma irradiation times.

Irradiation time (min)	β_{eff} ($\times10^3$cm MW^{-1})	Im$\chi^{(3)}$ ($\times10^{-6}$esu)	Is (MW cm^{-2})	ΔR	α_{NS}	n^2($\times10^{-13}$m^2 W^{-1})	$Re\chi^{(3)}$ ($\times10^{-6}$ esu)
0	-0.437±0.005	-3.36±0.04	3.6±0.2	0.192±0.005	0.100	-5.29±0.03	-1.587±0.009
2	-0.394±0.004	-3.03±0.03	5.4±0.4	0.203±0.004	0.101	-4.27±0.01	-1.281±0.003
3	-0.304±0.003	-2.34±0.02	9.9±0.5	0.187±0.003	0.098	-3.34±0.01	-1.002±0.003
5	-0.249±0.003	-1.91±0.02	12.8±0.6	0.185±0.002	0.099	-2.27±0.02	-0.681±0.005

Supplementary VI. Improvement of pulsed laser performance

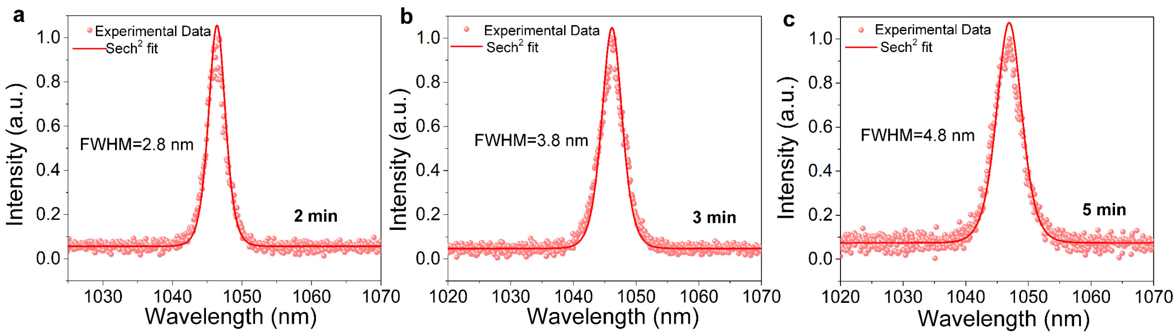

Supplementary Fig. 8 Improvement of pulsed laser performance. The spectrum of the mode-locked operation based on Bi_2O_2Se nanoplates under different plasma irradiation times. **a** 2 min. **b** 3 min. **c** 5 min. Their corresponding time bandwidths are 0.348, 0.353 and 0.349, respectively,

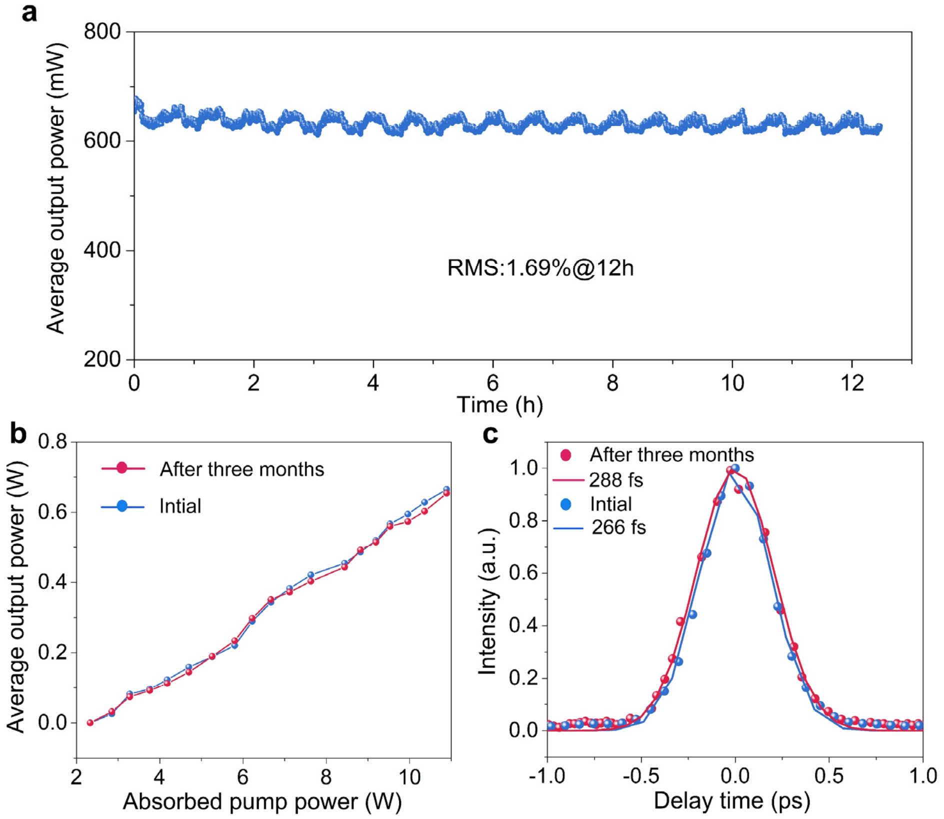

Supplementary Fig. 9 The stability of pulsed laser. a The stability measurements of mode-locked operations based on Bi_2O_2Se nanoplate SA. The average output power **b** and pulse width **c** of the CWML operation based on Bi_2O_2Se SA before and after exposure to air for three months.

Supplementary VII. Mode-locked laser at 2.0 μm

We employed a Bi_2O_2Se SA for all solid-state mode-locked lasers at 2.0 μm to further investigate its broadband response. The experimental setup is schematically shown in Fig. 2a. A 3×3×8 mm Tm:YAP crystal with 4% at. concentration was used as the gain medium. A fiber coupled laser diode emitting at 790 nm with a core diameter of 200 μm and a numerical aperture of 0.22 was used as the pump source. With a 1.8:1 optical collimation system, the pump spot radius focused into the crystal was ~56 μm, which was well matched with the radius of the TEM_{00} cavity mode (55 μm) calculated by ABCD propagation matrix theory. The oscillation laser mode radius on Bi_2O_2Se SA was 38 μm. The dichroic mirrors M1 (R=∞), M2 (R=0.8 m), and M3 (R=0.1 m) were all HR coated at 1.8-2.1 μm and high transmission (HT) coated at 780–810 nm. A flat output coupler (OC) with a transmission of 1% for a spectral range of 1.8-2.1 μm was used. Two Gires–Toutnois interferometer (GTI) mirrors with a total GDD of −600 fs^2 per round were used to compensate for the normal dispersion introduced by the crystal and Bi_2O_2Se SA.

With the Bi_2O_2Se SA used in the cavity, after careful adjustment, the laser runs into a continuous wave mode-locked (CWML) regime when the absorbed pump power exceeds 5.53 W, as shown in Fig. 10a. Under an absorbed pump power of 6.22 W, a maximum average output power of 51 mW is obtained. As shown in Fig. 10b, the output mode-locked laser spectrum is centered at 1941 nm with a full-width at half-maximum (FWHM) of 4.4 nm. Fig. 10c shows the pulse trains on the 200 ns and 500 μs time scales at the maximum output power, which indicates the realization of mode-locked laser operation. In addition, the recorded radio frequency spectrum is shown in Fig. S10d, with a fundamental beat note near 42.8 MHz and the corresponding signal-to-noise ratio of 52 dB, which is measured by a spectrum

analyzer (Agilent N9000A) with a resolution bandwidth (RBW) of 5 kHz. The inset of Fig. 10d is recorded over a wide span of 1 GHz with an RBW of 1.0 MHz, and the absence of any spurious modulation proves clean CWML operation at 2.0 μm based on Bi_2O_2Se SA. Due to the limitation of the measurement device (autocorrelator: Pulse Check 150) and at 2.0 μm, the mode-locked pulse duration is not measured, which can be deduced from the mode-locked FWHM (4.4 nm) and the time bandwidth product (0.315) to be ~900 fs. From the summarization of the mode-locked laser operation at 2.0 μm based on different nanomaterials (Table 3), it is obvious that the pulsed laser with Bi_2O_2Se nanoplates has a relatively shorter pulse width and higher average output power. In conclusion, the results of our experiments confirm that Bi_2O_2Se nanoplates can be used as an effective broadband saturable absorption material for pulse generation at 2 μm wavelengths.

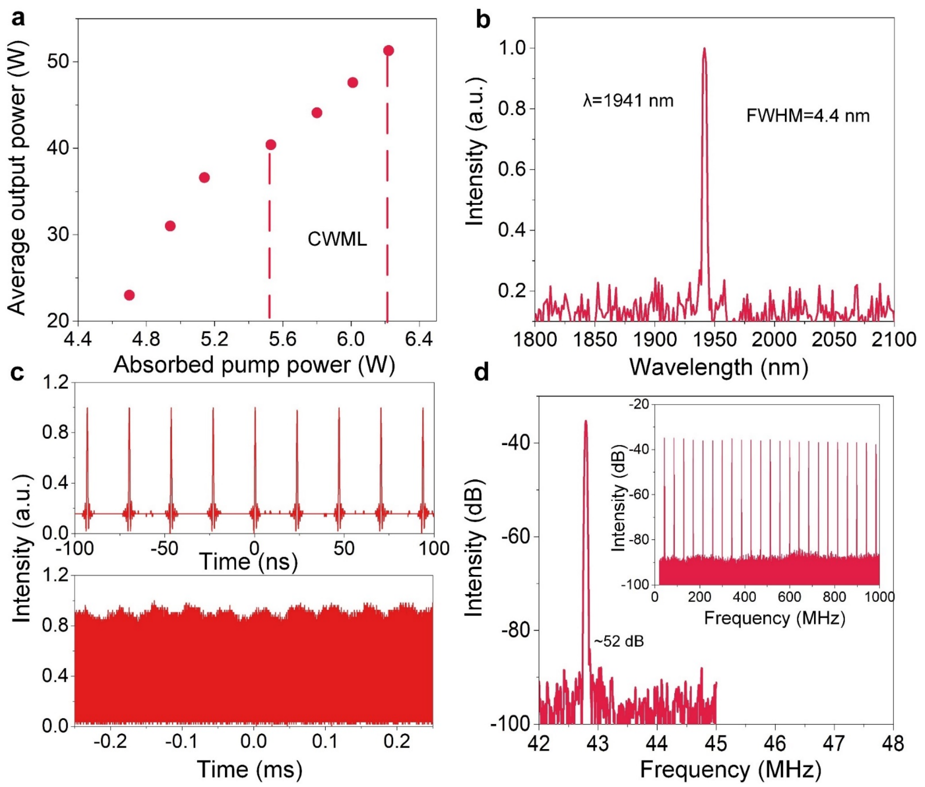

Supplementary Fig. 10 Mode-locked laser based on Bi_2O_2Se nanoplates at 2.0 μm. **a** Average output power of the CWML versus absorbed pump power. **b** Output mode-locked spectrum. **c** The pulse train with a span of 200 ns and 500 μs. **d** Recorded frequency spectrum with wide and narrow (inset) spans.

Supplementary Table 3. Results of mode-locked lasers operating at 2.0 μm based on various nanomaterials

Materials	Laser Gain Materials	Pulse Width (fs)	Output Power (mW)	Reference
Graphene	Tm^{3+}:CLNGG	729	60	8
SWCNTs	Tm^{3+}:Lu_2O_3	175	36	9
BP	Tm^{3+} silica fiber	793	1.5	10
MoS_2	Tm^{3+} silica fiber	1510	8	11
$MoTe_2$	Tm^{3+} silica fiber	952	36.7	12
$Ti_3C_2T_x$	Tm^{3+}-Ho^{3+}-codoped fiber	897	12.5	13
V_2C	Tm^{3+}-Ho^{3+}-codoped fiber	843	14	14
Bi_2O_2Se	Tm^{3+}:YAP	~1250	51	**This work**

Supplementary VIII References

1 Wu, J. X. et al. Controlled synthesis of high-mobility atomically thin bismuth oxyselenide crystals. *Nano Lett.* **17,** 3021-3026 (2017).

2 Chen, C. et al. Electronic structures and unusually robust bandgap in an ultrahigh-mobility layered oxide semiconductor, Bi_2O_2Se. *Science Adv.* **4,** 8355 (2018).

3 Pereira, A. L. J. et al. Experimental and theoretical study of Bi_2O_2Se under compression. *J. Phys. Chem. C* **122,** 8853-8867 (2018).

4 Khan, U. et al. Controlled Vapor Solid Deposition of Millimeter-Size Single Crystal 2D Bi_2O_2Se for High-Performance Phototransistors. *Adv. Funct. Mater.* **29**, 1807979 (2019).

5 Guo, J. et al. 2D GeP as a novel broadband nonlinear optical material for ultrafast photonics. *Laser Photonic. Rev.* **13,** 1900123 (2019).

6 Gao, L. F. et al. Ultrafast relaxation dynamics and nonlinear response of few-layer niobium carbide MXene. *Small Methods* **4,** 2000250 (2020).

7 Liu, D. et al. Giant nonlinear optical response of lead-free all-inorganic $CsSnBr_3$ nanoplates. *J. Phys. Chem. C* **125**, 803-811 (2021).

8 Ma, J. et.al. Graphene mode-locked femtosecond laser at 2 μm wavelength. *Opt. Lett.* **37,** 2085-2087 (2012).

9 Schmidt, A. et.al. 175 fs Tm:Lu_2O_3 laser at 2.07 μm mode-locked using single-walled carbon nanotubes. *Opt. Express* **20,** 5313-5318 (2012).

10 Sotor, J. et.al. Ultrafast thulium-doped fiber laser mode locked with black phosphorus. *Opt. Lett.* **40,** 3885-3888 (2015).

11 Cao, L. M.; Li, X.; Zhang, R.; Wu, D. D.; Dai, S. X.; Peng, J.; Weng, J.; Nie, Q. H., Tm-doped fiber laser mode-locking with MoS_2-polyvinyl alcohol saturable absorber. *Opt. Fiber Technol.* **41,** 187-192 (2018).

12 Wang, J. T. et.al. Ruan, S. C., Mode-locked thulium-doped fiber laser with chemical vapor deposited molybdenum ditelluride. *Opt. Lett.* **43,** 1998-2001 (2018).

13 Jhon, Y. I.; Lee, J.; Jhon, Y. M.; Lee, J. H., Ultrafast mode-locking in highly stacked $Ti_3C_2T_x$ MXenes for 1.9 μm infrared femtosecond pulsed lasers. *Nanophotonics* **10,** 1741-1751 (2021).

14 Lee, J.; Kwon, S. Y.; Lee, J. H., Investigation on the nonlinear optical properties of V_2C MXene at 1.9 μm. *J. Mater. Chem. C* **9,** 15346-15353 (2021).
